# Inhibition of autoimmune Th17 cell responses by pain killer ketamine

**DOI:** 10.18632/oncotarget.18324

**Published:** 2017-05-31

**Authors:** Jeong-Eun Lee, Jung-Man Lee, Young-Jun Park, Byung-Seok Kim, Young-Tae Jeon, Yeonseok Chung

**Affiliations:** ^1^ Laboratory of Immune Regulation, Institute of Pharmaceutical Sciences and College of Pharmacy, Seoul National University, Seoul, Republic of Korea; ^2^ BK21 plus program, College of Pharmacy, Seoul National University, Seoul, Republic of Korea; ^3^ Department of Anesthesiology and Pain Medicine, SMG-SNU Boramae Medical Center, Seoul, Republic of Korea; ^4^ Department of Anesthesiology and Pain Medicine, College of Medicine, Seoul National University, Seoul, Republic of Korea; ^5^ Department of Anesthesiology and Pain Medicine, College of Medicine, Seoul National University Bundang Hospital, Seongnam, Republic of Korea

**Keywords:** ketamine, Th17 cell, STAT3, IL-21, autoimmunity, Immunology and Microbiology Section, Immune response, Immunity

## Abstract

Ketamine is widely used in animals and humans as a systemic anesthetic. Although several immune-modulatory functions of ketamine have been reported, the effects of ketamine on the differentiation of Th17 cell are unknown. We found that ketamine significantly diminished the frequency of IL-17-producers among CD4^+^ T cells stimulated under Th17-skewing conditions. Mechanistic studies showed that ketamine had little effect on the production of Th17-inducing cytokines by dendritic cells and the proliferation of T cells in response to anti-CD3; however it significantly hampered IL-21 expression as well as STAT3 phosphorylation in T cells upon IL-6 stimulation. Moreover, MOG-reactive CD4^+^ T cells expanded in the presence of ketamine produced reduced amounts of Th17 cytokines, leading to diminished EAE severity when transferred into TCRβ-deficient mice in comparison to those treated with vehicle. These findings demonstrate that ketamine suppresses autoimmune Th17 cell responses by inhibiting the differentiation as well as the reactivation of Th17 cells.

## INTRODUCTION

Immuno-modulatory effects of various anesthetics have been extensively studied in recent years. For example, volatile anesthetics have been proven to increase tumor formation, whereas propofol has been shown to exert protective effects in preclinical studies [1]. In addition, sevoflurane has been reported to inhibit T cell activation and ameliorate clinical symptoms of experimental autoimmune encephalomyelitis (EAE), an animal model of human multiple sclerosis [2]. When used as an anesthetic in patients during surgery, propofol exerts an immuno-protective role by promoting Th1 cell differentiation [3]. Lidocaine is another example that has been reported to inhibit Th1 cell response by suppressing the secretion of pro-inflammatory cytokines in dendritic cells [4].

Ketamine is widely used as an anesthetic and analgesic for general anesthesia and chronic pain management [5]. The primary mechanism of action of ketamine is through the noncompetitive antagonism of the N-methyl D-aspartic acid (NMDA) receptor, releasing tonic inhibition from cortical γ-aminobutyric acid-ergic (GABAergic) interneurons on output neurons [6]. By stimulating the mTOR signaling pathway, ketamine can also prolong anti-depressant effects via the increased connectivities of neuronal synapses [7]. In addition to its well-known function as an anesthetic, ketamine has been shown to exert immuno-regulatory effects. For instance, ketamine inhibited the priming of Th1-mediated immune response by suppressing maturation of bone marrow-derived dendritic cells (BMDC) [8]. However, its effects on other Th subsets including Th17 remain elusive.

IL-17-producing T helper cell subset (Th17) has emerged as a crucial player in the adaptive immune system during the last decade [9]. Th17 cell differentiation is initiated by the activation of STAT3 in the presence of IL-6 and transforming growth factor-β (TGF-β) [10], which induces the transcription of Th17 cell-associated genes, including *Rorc, Il17* and *Il23r*. While TGF-β and IL-6 drive the initial stages of Th17 cell lineage commitment, IL-21 produced by Th17 cells acts as an autocrine factor that further promotes Th17 cell differentiation by amplifying STAT3 activation through a positive feedback loop. On the other hand, IL-23 plays a critical role in the terminal differentiation and maintenance of Th17 cell lineage through inducing Blimp1 expression in Th17 cells [11, 12]. The association of Th17 cells with autoimmune disorders in humans has been demonstrated in various diseases including multiple sclerosis, rheumatoid arthritis, inflammatory bowel disease and psoriasis. Indeed, several neutralizing or blocking antibodies targeting Th17/IL-17 pathway have shown clinical efficacy in plaque psoriasis [13–15]. Moreover, development of small molecules targeting STAT3-RORγt pathway is an active area of new drug development worldwide.

In this study, we aimed to characterize the immuno-modulatory effects of ketamine on autoimmune Th17 cell responses. Our findings indicate that ketamine regulates Th17 cell differentiation through the inhibition of autocrine IL-21 production and STAT3 phosphorylation in T cells. As a result, ketamine treatment significantly reduced the pathogenicity of myelin-reactive Th17 cells in a passive EAE model, suggesting a suppressive role in autoimmune Th17 cell responses.

## RESULTS

### Ketamine suppresses dendritic cell-mediated Th17 cell differentiation

**Figure 1:**
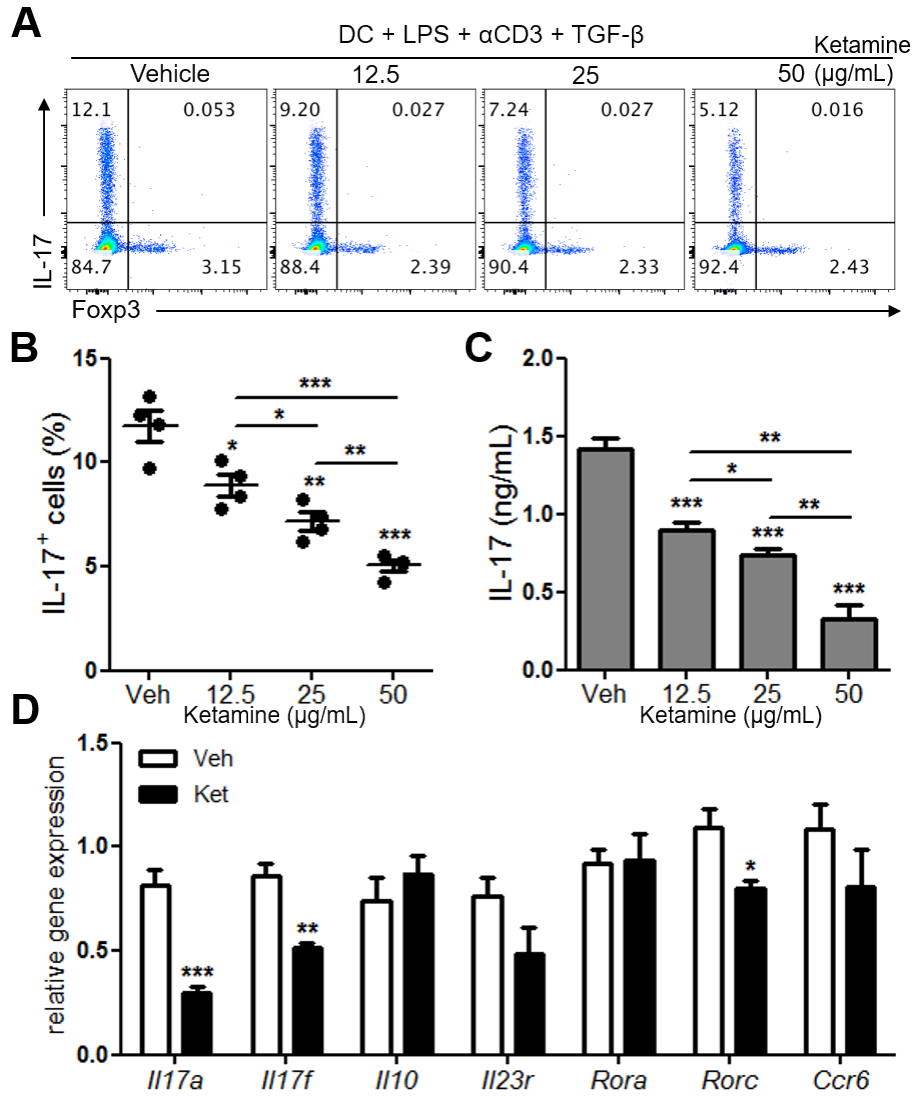
Ketamine inhibits DC-mediated Th17 cell differentiation Naïve CD4^+^ T cells and CD11c^+^ bone marrow-derived dendritic cells were stimulated with soluble anti-CD3 and co-cultured under Th17-skewing condition for 3 days. Detection of IL-17 expression cells was conducted using flow cytometry analysis. **A.**, **B.** The levels of IL-17 in the supernatant were determined by ELISA. **C.** The expression levels of indicated transcripts were analyzed by quantitative real-time RT-PCR. **D.** Data represent three independent experiments. Data shown are mean ± SEM. *, *p* < 0.05, **, *p* < 0.01, ***, *p* < 0.001.

Stimulation of naïve CD4^+^ T cells with dendritic cells in the presence of anti-CD3, LPS and TGF-β induces Th17 differentiation [10]. As a first step in examining the effects of ketamine on Th17 cell responses, we determined if ketamine impacts dendritic cell-mediated Th17 cell differentiation *in vitro*. Compared with vehicle-treated condition, addition of ketamine significantly decreased the frequency of IL-17-expressing CD4^+^ T cells in a dose dependent manner, while that of Foxp3-expressing cells remained comparable (Figure 1A & 1B). The amount of IL-17 in the culture supernatant was also remarkably decreased by ketamine treatment in a dose-dependent manner (Figure 1C). Moreover, the levels of *Il17a, Il17f, Rorc* were all significantly decreased by ketamine treatment compared to vehicle treatment (Figure 1D). The levels of *Il23r, Ccr6* were also slightly decreased, while that of *Rora* remained unchanged by ketamine treatment. These results together demonstrate that ketamine inhibited dendritic cell-mediated differentiation of naïve T cells into Th17 cells.

### Ketamine suppresses Th17 cell differentiation in a T cell-intrinsic manner

**Figure 2:**
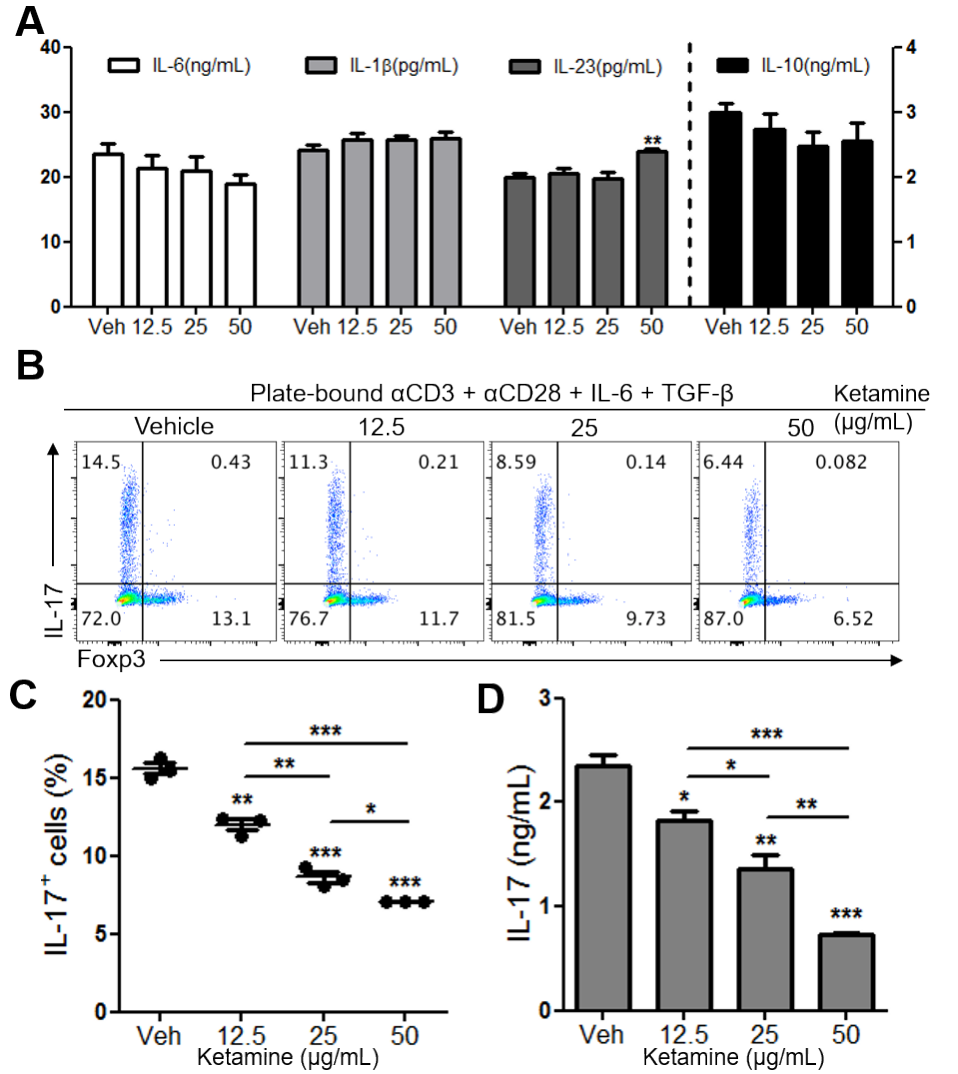
Effect of ketamine on DCs and CD4^+^ T cells during Th17 cell differentiation Bone marrow-derived dendritic cells were stimulated with 100 ng/mL of LPS in the presence of various concentrations of ketamine for 24 hours. The amounts of indicated cytokines in the supernatant were measured by ELISA. **A.**. FACS-sorted naïve CD4^+^ T cells were stimulated with plate-bound anti-CD3 and anti-CD28 under Th17-skewing condition for 3 days, and the frequency of IL-17-expressing T cells were analyzed. **B.**, **C.** IL-17 concentrations of the supernatants were measured by ELISA. **D.** Data represent at least 3 independent experiments. Data shown are mean ± SEM. *, *p* < 0.05, **, *p* < 0.01, ***, *p* < 0.001.

Ketamine has been shown to modulate the function of dendritic cells [8]. Therefore, we asked if the observed suppression of Th17 cell differentiation by ketamine was due to the decreased production of Th17-inducing cytokines, such as IL-6, IL-1β and IL-23 [16], from dendritic cells. To this end, we stimulated DCs with LPS in the presence or absence of ketamine for 24 hours before measuring the production of Th17-inducing cytokines. As depicted in Figure 2A, the concentrations of IL-1β, IL-6 and IL-23 between vehicle- and ketamine-treated conditions were largely comparable, indicating that ketamine had little role in the production of Th17 cell-promoting cytokines by dendritic cells. Similarly, the production of IL-10 from LPS-stimulated dendritic cells was not affected by ketamine treatment (Figure 2A).

This observation prompted us to ask if ketamine inhibits Th17 cell differentiation in a T cell-intrinsic manner. To test this hypothesis, we stimulated naïve CD4^+^ T cells with plate-bound anti-CD3 and anti-CD28 under Th17-skewing condition (IL-6 + TGF-β [10]) in the presence of ketamine or vehicle. Notably, we observed a significant reduction in the frequency and number of IL-17-producing CD4^+^ T cells by ketamine in a dose-dependent manner (Figure 2B & 2C, Supplementary Figure 1A). Consistently, the amount of IL-17 in the supernatant was also remarkably decreased by ketamine treatment (Figure 2D). The frequency of apoptotic cells among T cells was comparable between vehicle- and ketamine-treated groups (Supplementary Figure 1B). Moreover, the reduction of Th17 cell frequency did not result in the increase of Foxp3^+^ regulatory T cells regardless of Th17 cell differentiation condition (Supplementary Figure 1C & D). Considering the role of TGF-β in inducing Foxp3^+^ Treg cells [17], this indicates that the inhibition of Th17 cell differentiation by ketamine was not due to the increase of Foxp3^+^ Treg cells in this experimental setting. Collectively, these results demonstrate that ketamine induced the inhibition of Th17 cell differentiation in a T cell-intrinsic manner rather than through the modulation of dendritic cells.

### Effects of ketamine on the proliferation of CD4^+^ T cells

**Figure 3:**
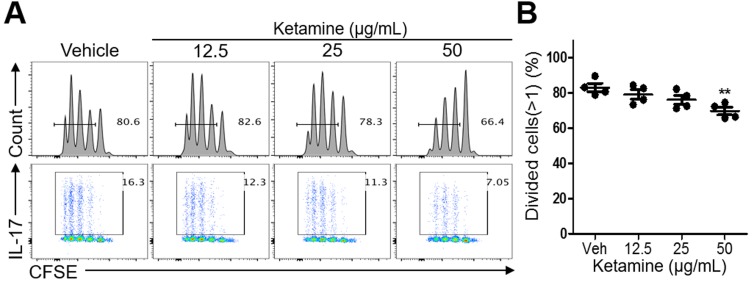
Effect of ketamine on the proliferation of T cells during Th17 cell differentiation Naïve CD4^+^ T cells were labeled with CFSE before being stimulated with plate-bound anti-CD3 and anti-CD28 under Th17-skewing condition for 3 days. The dilution of CFSE and the frequency of IL-17-expressing cells were analyzed by flow cytometry. **A.** The proportion of cells divided more than once was measured. **B.** Data represent two independent experiments. Data shown are mean ± SEM. *, *p* < 0.05, **, *p* < 0.01, ***, *p* < 0.001.

To determine the mechanism by which ketamine inhibits Th17 cell differentiation, we asked if ketamine impacts the activation and proliferation of CD4^+^ T cells in response to anti-CD3-mediated stimulation. Naïve CD4^+^ T cells were labeled with CFSE dye before being stimulated with anti-CD3 and anti-CD28 under Th17-skewing condition. Of note, the frequency of cells that divided more than once was comparable between vehicle-treated and ketamine-treated T cells at the 12.5, and 25 μg/ml doses (Figure 3A & 3B). The proliferation of T cells was, however, slightly, but significantly, decreased by ketamine at a higher dose (50 μg/ml).

### Ketamine suppresses the phosphorylation of STAT3 and the expression of IL-21 in CD4^+^ T cells upon IL-6 stimulation

**Figure 4:**
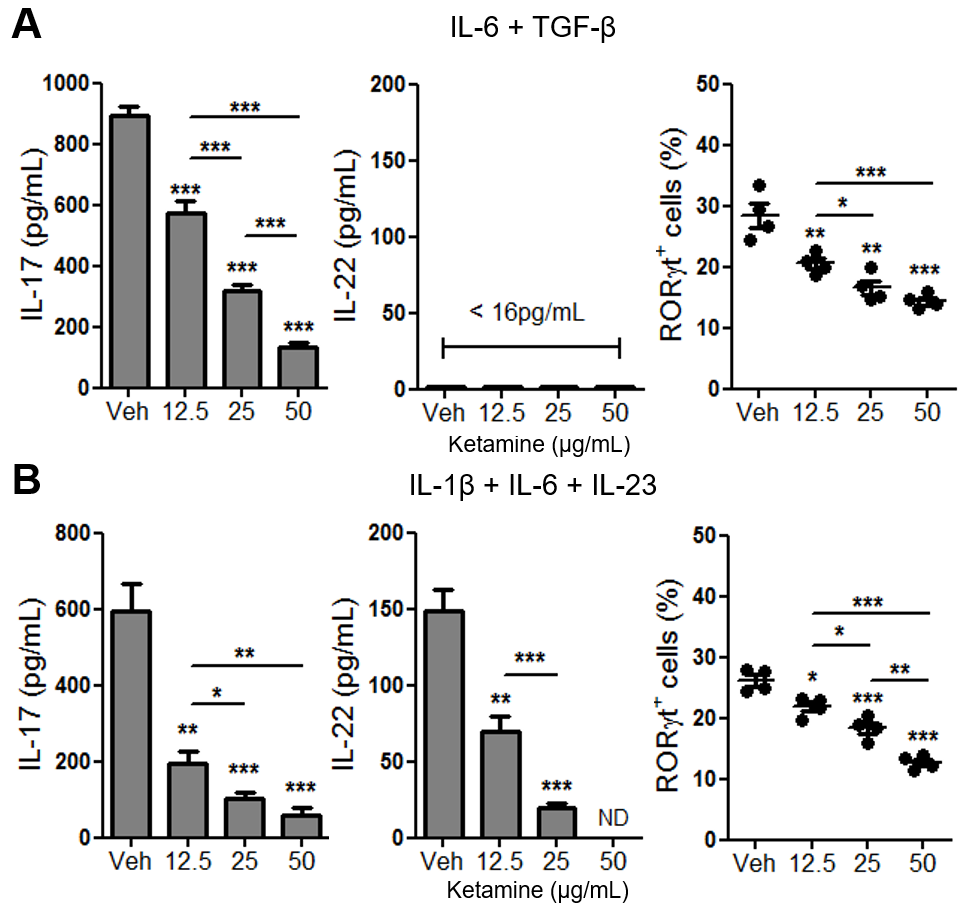
Ketamine inhibits Th17 cell differentiation induced by IL-6, IL-1β and IL-23 in the absence of exogenous TGF-β Naïve CD4^+^ T cells were stimulated with plate-bound anti-CD3 and anti-CD28 in the presence of IL-6 plus TGF-β. **A.** or IL-1β plus IL-6 plus IL-23 **B.** for 3 days. The levels of IL-17 and IL-22 in the supernatant as well as the frequency of RORγt-expressing T cells were analyzed by ELISA and flow cytometry, respectively. ND, Not detectable. Data represent two independent experiments. Data shown are mean ± SEM. *, *p* < 0.05, **, *p* < 0.01, ***, *p* < 0.001.

Stimulation of naïve CD4^+^ T cells with IL-6 and TGF-β efficiently induces the expression of RORγt and IL-17, but not IL-22, by T cells [10] (Figure 4A). On the other hand, stimulation of T cells with IL-1β, IL-6 plus IL-23 in the absence of exogenous TGF-β is also known to trigger the differentiation of Th17 cells that produce IL-22 [18, 19]. We sought to determine whether ketamine also inhibits the differentiation of Th17 cells induced by IL-1β, IL-6 plus IL-23, and found that addition of ketamine significantly suppressed the production of IL-17, as well as IL-22 from T cells in a dose dependent manner (Figure 4B). The magnitude of IL-17 suppression by ketamine appeared to be more significant in the IL-6, IL-23 and IL-1β stimulation condition (Figure 4A & 4B). The level of transcription factor RORγt in T cells was also significantly decreased by ketamine treatment (Figure 4A & 4B).

**Figure 5:**
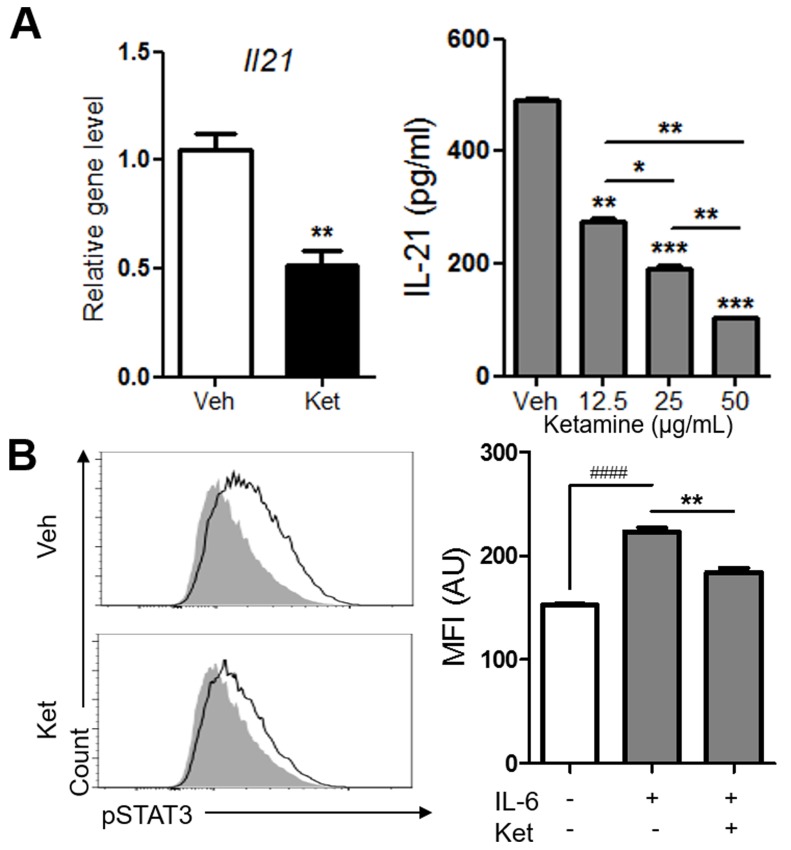
Ketamine negatively regulates the expression of IL-21 and the phosphorylation of STAT3 during Th17 cell differentiation Naïve CD4^+^ T cells were stimulated with plate-bound anti-CD3 and anti-CD28 under Th17-skewing condition for 48 hours in the presence or absence of ketamine. The level of *Il21* transcript as well as IL-21 in the supernatant was determined. **A.** The levels of phosphorylated STAT3 in CD4^+^ T cells stimulated under plate-bound anti-CD3 and anti-CD28 plus IL-6 conditions were measured by flow cytometry. **B.** Shaded, IL-6 unstimulated negative control; closed line, vehicle or ketamine treated; NC. Data represent two independent experiments. Data shown are mean ± SEM. *, *p* < 0.05, **, *p* < 0.01, ***, *p* < 0.001, ####, *p* < 0.0001.

Since IL-6 was the only common cytokine in the two different Th17 cell differentiation conditions inhibited by ketamine, we reasoned that ketamine suppresses Th17 cell differentiation probably by inhibiting IL-6 signaling pathway. IL-6 signals through STAT3 to induce autocrine IL-21 secretion in CD4^+^ T cells during Th17 cell differentiation [20, 21]. Of interest, ketamine significantly decreased the mRNA transcription and protein production of IL-21 from T cells stimulated with IL-6 in the presence of anti-CD3 (Figure 5A). To further explore the molecular mechanism, we determined the involvement of STAT3 and found that the level of phosphorylated STAT3 in T cells induced by IL-6 was significantly diminished by ketamine treatment (Figure 5B). The mean fluorescence intensity of phosphorylated STAT3 was dramatically decreased by ketamine compared to vehicle-treated cells with p value of 0.0025 (Figure 5B). Collectively, these data demonstrate that ketamine suppresses Th17 cell differentiation program by inhibiting the phosphorylation of STAT3 upon IL-6 stimulation and subsequent expression of IL-21 in T cells.

### Ketamine suppresses autoimmune Th17 cells

**Figure 6:**
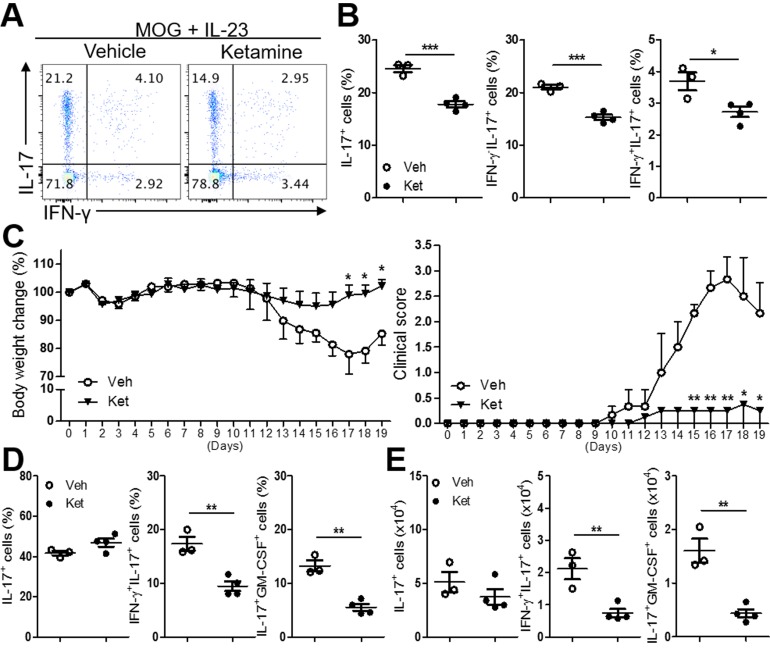
Ketamine inhibits the reactivation of MOG-reactive Th17 cells Lymphoid cells from the MOG-immunized mice were re-stimulated with MOG peptide plus IL-23 in the presence of ketamine or vehicle for 5 days before CD4^+^ T cells were sorted by MACS. The expression of IL-17 and IFN-γ was measured. **A.**, **B.** The sorted CD4^+^ T cells were i.v. transferred into TCRβ-deficient mice and the recipient mice were s.c. injected with MOG in CFA. Body weight and clinical disease score were daily monitored **C.**, and the frequencies and absolute numbers of the indicated population among CD4^+^ T cells in the CNS of the recipients were determined **D.** & **E.** Data represent two independent experiments. Data shown are mean ± SEM. *, *p* < 0.05, **, *p* < 0.01, ***, *p* < 0.001.

Since Th17 cells are known to mediate tissue inflammation during autoimmune diseases, we next asked if ketamine also impacts autoimmune Th17 cell responses. Immunization with myelin oligodendrocyte glycoprotein (MOG_35-55_) peptide induces the generation of autoreactive T cells that can trigger the onset of experimental autoimmune encephalomyelitis (EAE) in mice [22]. We immunized C57BL/6 mice with MOG in CFA, and isolated lymphoid cells from the draining lymph nodes, and stimulated them with MOG in the presence of IL-23, which triggers the expansion of MOG-reactive autoimmune Th17 cells [23]. Addition of ketamine remarkably decreased the frequency of IL-17-producing cells among CD4^+^ T cells (Figure 6A & 6B), indicating the inhibitory effect of ketamine on the expansion of autoimmune Th17 cells. By contrast, little change in the frequency of Foxp3^+^ T cells by ketamine treatment was observed in this experimental setting (Supplementary Figure 2).

When these cells were adoptively transferred into *Tcrb*^-/-^ recipient mice, the recipients of ketamine-treated T cells exhibited significantly less severe EAE (max clinical score: 2.83 ± 0.44 vs. 0.25 ± 0.25, *p* = 0.0028) associated with less severe loss of body weight in comparison with the recipients of vehicle-treated T cells (Figure 6C). Analysis of the CD4^+^ T cells isolated from brain and spinal cord showed a significant decrease in IFN-γ^+^IL-17^+^ as well as GM-CSF^+^IL-17^+^ T cells in the recipients of ketamine-treated T cells (Figure 6D & 6E). These results indicate that the differentiation of MOG-reactive IL-17^+^ T cells into pathogenic IFN-γ^+^IL-17^+^ T cells *in vivo* was significantly attenuated by ketamine treatment, although the frequency of total IL-17^+^ T cells remained unaffected. Collectively, these results demonstrate that ketamine inhibited the expansion and/or reactivation of pathogenic autoimmune Th17 cell responses.

## DISCUSSION

Considering the critical contribution of immune cells to wound healing and host defense [24], it seems reasonable to surmise that the immune-modulatory functions of anesthetic agents affect the postoperative recovery in patients [25]. In this regard, ketamine is known to suppress Th1 cell-mediated immune responses and to inhibit the maturation and activation of dendritic cells [8]. Ketamine has also been shown to decrease IL-6 and TNF-α in human peripheral blood mononuclear cells (PBMCs) [26]. Accumulating evidence demonstrates a crucial pathogenic role of Th17 cells in mediating autoimmune diseases in humans [27, 28]. However, no studies to date have addressed if ketamine plays any role on Th17 cell responses and Th17 cell-mediated immune diseases.

In the present study, we unveil that ketamine negatively regulates the differentiation and reactivation of Th17 cells. Although it has been reported that ketamine inhibits Th1 differentiation through suppressing IL-12p40 production by dendritic cells [8], ketamine had little role in the production of Th17-promoting cytokines including IL-6, IL-23 and IL-1β by DCs, strongly suggesting that ketamine directly impacts T cells. Indeed, we observed that ketamine treatment significantly inhibited Th17 cell differentiation in DC-free Th17-skewing conditions. In the CFSE-dilution assay, we observed little effect of ketamine on the proliferation of naïve CD4^+^ T cells in response to anti-CD3. Instead, ketamine suppressed the differentiation of naïve T cells into Th17 cells by inhibiting IL-6-mediated STAT3 phosphorylation, leading to a significant decrease in the expression of IL-21 and RORγt in T cells. Upon IL-6 stimulation, phosphorylated STAT3 in T cells induce multiple Th17-related genes, including *Il17, Il21, Rorc, Irf4* and *Ahr* [29]. Since STAT3 is a crucial factor for IL-21 induction, as well as one of the downstream mediators of the IL-21 signaling pathway, it is unclear whether the decrease in IL-21 production from T cells by ketamine was the result of, or the cause of the reduced STAT3 phosphorylation in T cells. It is likely that diminished phosphorylation of STAT3 resulted in decreased expression of IL-21 in T cells, which further prolonged the decreased STAT3 phosphorylation. Further studies will be needed to determine the molecular mechanism by which ketamine suppresses STAT3 phosphorylation in T cells.

In addition to its inhibitory role in early Th17 cell differentiation, ketamine was also found to suppress the reactivation of MOG-specific Th17 cells upon IL-23 stimulation. Upon transfer into lymphopenic recipient mice, MOG-specific Th17 cells expanded in the presence of ketamine were less pathogenic in inducing EAE in the recipients in comparison to those expanded in the presence of vehicle. Analysis of CD4^+^ T cells in the CNS revealed a significant reduction in the frequencies of IFN-γ^+^IL-17^+^ cells and GM-CSF^+^IL-17^+^ cells in the former group, both of which play a critical role in Th17-mediated CNS inflammation. These results strongly suggest that ketamine significantly impairs the terminal differentiation of autoimmune Th17 cells into pathogenic IFN-γ^+^IL-17^+^ cells and GM-CSF^+^IL-17^+^ cells. Since IL-23 also signals through STAT3 activation [30], it is possible to surmise that ketamine also suppresses IL-23-mediated phosphorylation of STAT3 in Th17 cells during reactivation. IL-23 induces the expression of Blimp1 to stabilize the Th17 cell lineage program [31]. Thus, it is also possible that ketamine inhibits the induction of Blimp1 expression upon IL-23 stimulation in Th17 cells. The exact mechanism by which ketamine suppresses the reactivation of Th17 cells is, however, unclear at this stage.

In summary, the present study unveils a strong inhibitory effect of ketamine on Th17 cell responses at two different stages, differentiation and reactivation. The strength of this study is the possibility of clinical applicability. Besides the treatment of chronic pain, ketamine or its derivatives can be further considered as a novel therapeutic approach for Th17 cell-mediated diseases in humans such as psoriasis [32] and rheumatoid arthritis [33]; however, additional mechanistic and translational studies will be needed before applying our findings into clinical settings in humans.

## MATERIALS AND METHODS

### Ethics approval

All mouse experiments were performed as approved by Seoul National University Institutional Animal Care and Use Committee (IACUC, Seoul National University approved protocol #SNU- 160422-3-1).

### Mice

C57BL/6 mice within the ages of 6-10 weeks were purchased from Orient Bio (Gyeonggi, Republic of Korea). *Tcrb*^-/-^ mice were purchased from Jackson Laboratories (Maine, USA). Mice were maintained under semi-specific-pathogen-free animal facility in sterile, individually ventilated cages at the Seoul National University with free access to sterile water. CO_2_ inhalation was used for euthanasia.

### *In vitro* T cell differentiation

CD4^+^ T cells were isolated from C57BL/6 mice were enriched by CD4 microbeads (Miltenyi Biotec), followed by sorting CD4^+^CD25^-^CD62L^hi^CD44^lo^ cells using the FACSARIA III (BD Biosciences). For DC:T co-culture experiments, sorted naïve CD4^+^ T cells (1.0 × 10^5^ cells/well) were co-cultured with CD11c^+^ bone marrow-derived DCs (1.0 × 10^4^ cells/well) in the presence of soluble anti-CD3 (0.3 μg/mL), LPS (100 ng/mL), and TGF-β (5 ng/mL). For plate-bound Th17 cell differentiation experiments, sorted naïve CD4^+^ T cells (1.0 × 10^5^/well) were stimulated with plate-bound anti-CD3 and anti-CD28 in the presence of IL-6 (20 ng/mL), and TGF-β (5 ng/mL) or IL-6 (40 ng/mL), IL-1β (10 ng/mL), and IL-23 (50 ng/mL). The concentrations of treated ketamine (Ketamine hydrochloride, Yuhan Co., Korea) were 12.5, 25, 50μg/mL and sterile water was used as vehicle.

### Flow cytometry for cytokine analysis

Cells cultured or obtained from mice were incubated for 4 hours with Phorbol 12-myristate 13-acetate (PMA) (100 ng/mL, Sigma, St Louis, MO, USA) and ionomycin (1 μM, Sigma, St Louis, MO, USA) in the presence of Brefeldin A and Monensin (eBioscience, San Diego, CA, USA). The stimulated T cells were treated in Fix/Permeabilization buffer. PE- or APC-conjugated anti-IL-17A (Biolegend), PerCP/Cy5.5-conjugated anti-IFN-γ (Biolegend), PE-conjugated anti-RORγt, eFlour 450-conjugated anti-Foxp3 (eBioscience), Alexa Flour 488-conjugated anti-STAT3 phospho(Tyr705) (Biolegend) were used for intracellular staining. To analyze CNS-infiltrating immune cells, lymphoid cells from CNS were stained with PE/Cy7-conjugated anti-CD4 (eBioscience), PE-conjugated anti-GM-CSF (Biolegend), PerCP/Cy5.5-conjugated anti-IFN-γ (Biolegend), and Alexa Flour 488-conjugated anti-IL-17A (Biolegend). Samples were analyzed using the FACSVerse flow cytometer (BD Bioscience, San Jose, CA) and data were analyzed through the FlowJo software (TreeStar, Ashland, OR) [34].

### Cytokine ELISA

The amounts of IL-1β, IL-6, IL-21, IL-23, IL-17 and IL-22 cytokines in the culture supernatant were measured using an ELISA kit (Biolegend and eBioscience). All assays were performed according to the manufacturer's protocol. For IL-17 detection, samples were obtained after 3-4 days of T cell differentiation. For IL-21 detection by IL-6 stimulation, cells were stimulated by IL-6 for 48 hours and re-stimulated by anti-CD3 coated condition after normalization to 1.0 × 10^5^ cells/well in 96-well culture plate. For IL-1β, IL-6, IL-10, IL-23 detection from dendritic cells, cultured BMDCs were purified by CD11c^+^ micro-bead before being treated with indicated conditions for 24 hours.

### Quantitative real-time PCR

Total RNA was obtained from *in vitro* cultured CD4^+^ T cells with TRIzol (Invitrogen). Complementary DNA was synthesized with amfiRivert reverse transcriptase (GenDEPOT) and the levels of indicated mRNA transcript were quantified by iTaq Universal SYBR Green Supermix (Bio-Rad Laboratories) with ABI-PRISM 7900 detection system (Applied Biosystems). All the data were normalized to the expression of *Gapdh*. The primer pair for *Gapdh* was: forward, 5’-GAGAACTTTGGCATTGTGG-3’, reverse, 5’-ATGCAGGGATGATGTTCTG-3’. The primer pairs of *Il17a*, *Il17f*, *Il10,* *Il21*, *Il23r*, *Rora*, *Rorc* and *Ccr6* were previously described [35]. The concentration of treated ketamine (Ketamine hydrochloride, Yuhan Co., Korea) was 50 μg/mL and sterile water was used as vehicle.

### *Ex vivo* re-stimulation of MOG-reactive T cells and *in vivo* CD4^+^ T cell transfer EAE

C57BL/6 mice were subcutaneously immunized with 100 μl mix of 300 μg of MOG_35-55_ and 100 μg of CFA (Sigma Aldrich) containing killed *M. tuberculosis* (5 mg/ml) for 7-8 days [35]. After immunization period, lymphoid cells from the inguinal lymph nodes were stimulated with MOG_35-55_ (20 μg/mL) in the presence of IL-23 (20 ng/mL), anti-IFN-γ (XMG1.2, 10 μg/mL) plus vehicle (sterile water) or ketamine (50 μg/mL) for 5 days. The expression of IL-17 and IFN-γ was analyzed by flow cytometry. For *in vivo* CD4^+^ T cell transfer EAE study, the re-stimulated CD4^+^ T cells were purified intravenously transferred to *Tcrb*^-/-^ recipient mice (0.6-2×10^5^ cells) (day 0). On day 1, the recipients were immunized with MOG_35-55_ in CFA and were injected with pertussis toxin on day 2. Body weight and clinical score were monitored daily.

### Statistics

Data were analyzed by using the GraphPad Prism 5 (GraphPad Software). The Student's unpaired two-tailed t-test with 95% of confidence intervals was applied for statistical analyses. Data shown are presented in mean ± SEM and p values were calculated with *t*-test.

## SUPPLEMENTARY MATERIALS FIGURES


