# Supplementary material for: Inhibition of autoimmune Th17 cell responses by pain killer ketamine
Source: Oncotarget. 2017 May 31;8(52):89475–85. doi: 10.18632/oncotarget.18324 (PMC5685685; doi:10.18632/oncotarget.18324)
Supplement: Supplementary file 1 [file oncotarget-08-89475-s001.pdf]

Inhibition of autoimmune Th17 cell responses by pain killer ketamine

Supplementary Material

METHODS

Flow cytometry for Annexin V assay

Cells were harvested after 3 days of differentiation. APC-conjugated Annexin V (Biolegend) was used for apoptosis assay. Samples were analyzed using the FACSVerse flow cytometer (BD Bioscience, San Jose, CA) and data were analyzed through the FlowJo software (TreeStar, Ashland, OR).

Ex vivo re-stimulation of MOG-reactive T cells

Immunization experiment is described in main method section. After immunization period, lymphoid cells from the inguinal lymph nodes were stimulated with $MOG_{35\text{-}55}$ (20 µg/mL) in the presence of IL-23 (20 ng/mL) plus vehicle (sterile water) or ketamine (12.5, 25, 50 µg/mL) for 5 days. The expressions of IL-17 and Foxp3 were analyzed by flow cytometry.

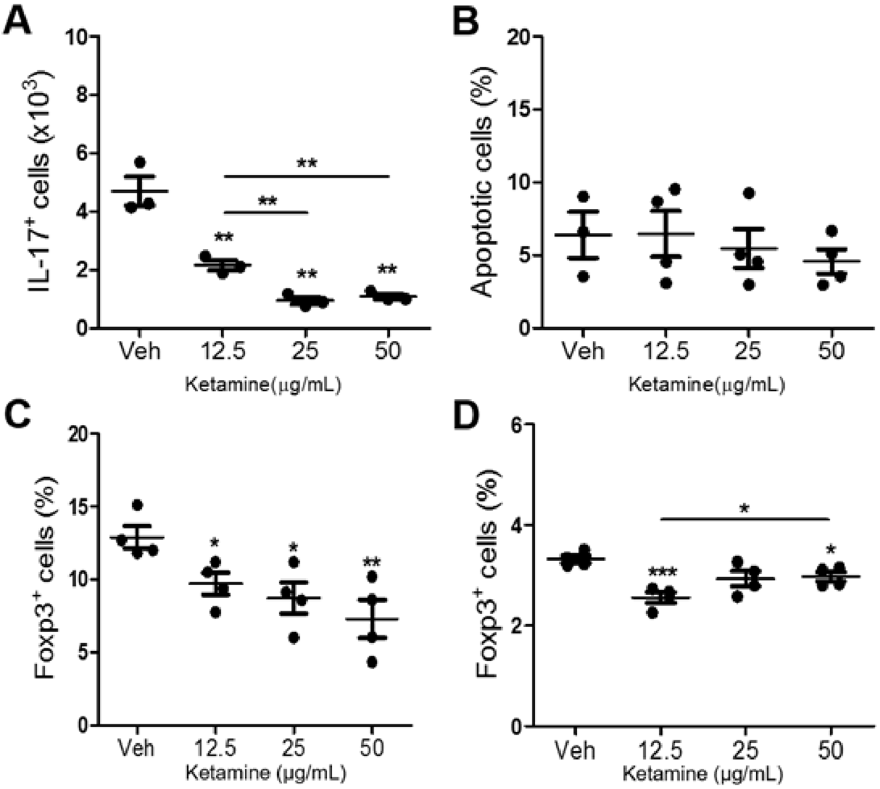

Supplementary Figure 1: Effects of ketamine on the expression of IL-17 and Foxp3 and on apoptosis of T cells. FACS-sorted naïve CD4+ T cells were stimulated with plate-bound anti-CD3 and anti-CD28 under Th17-skewing condition for 3 days. The absolute cell number of IL-17-expressing T cells were analyzed (A). The frequency of Annexin V+ apoptotic cells (B) and Foxp3-expressing T cells (C) were analyzed. Naïve CD4+ T cells and CD11c+ bone marrow-derived dendritic cells were stimulated with soluble anti-CD3 and co-cultured under Th17-skewing condition for 3 days and the frequency of Foxp3-expressing cells was analyzed (D). Bars shown are mean ± SEM. *, $p<0.05$, **, $p<0.01$, ***, $p<0.001$.

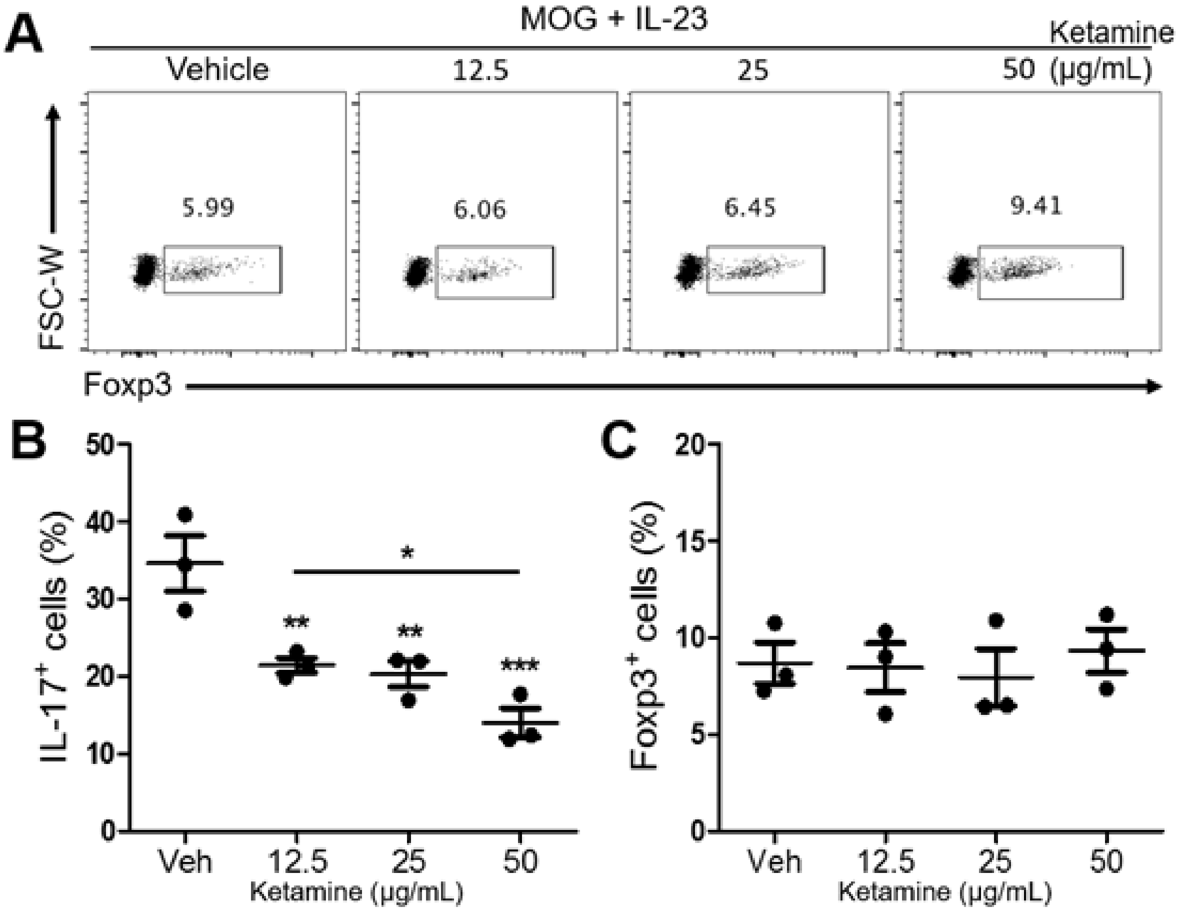

Supplementary Figure 2: Ketamine inhibits the reactivation of MOG-reactive Th17 cells. Lymphoid cells from the MOG-immunized mice were re-stimulated with MOG peptide plus IL-23 in the presence of ketamine or vehicle for 5 days before $CD4^+$ T cells were sorted by MACS. The expressions of IL-17 and Foxp3 were analyzed using flow cytometry analysis (A-C). Bars shown are mean ± SEM. *, p<0.05, **, p<0.01, ***, p<0.001.
